# Analyzing the Evolution of Medical Ethics Education: A Bibliometric Analysis of the Top 100 Cited Articles

**DOI:** 10.7759/cureus.41411

**Published:** 2023-07-05

**Authors:** Sakshi Roy, Muhammad Hamza Shah, Arjun Ahluwalia, Amer Harky

**Affiliations:** 1 School of Medicine, Dentistry and Biomedical Sciences, Queen’s University Belfast, Belfast, GBR; 2 Cardiothoracic Surgery, Liverpool Heart and Chest Hospital, Liverpool, GBR

**Keywords:** scopus, bibliometric analysis, ethics education, medical ethics, medical education

## Abstract

Ethics education plays a pivotal role in healthcare by providing professionals and students with the essential competencies to navigate intricate ethical challenges. This study conducts a comprehensive bibliometric analysis of the most-cited articles on ethics education, investigating parameters such as citation count, document types, geographical origin, journal analysis, publication year, author analysis, and keyword usage. The findings reveal a substantial impact characterized by high citation counts and the influence of a prominent publication focusing on the hidden curriculum and structure of medical education. Moreover, the analysis demonstrates a discernible increase in research output since 2000, signaling a growing recognition of the significance of ethics education in the healthcare domain. Notably, specific journals, particularly those dedicated to medical education and ethics, emerge as major contributors in this field, publishing many articles. Renowned authors have made noteworthy contributions, and emerging themes encompass the ethical implications of virtual reality and artificial intelligence in healthcare education. Additionally, undergraduate medical education garners significant attention, emphasizing the importance of establishing ethical values and professionalism early. Overall, this study highlights the imperative of interdisciplinary collaboration and the necessity for effective ethics education programs to equip healthcare professionals with the requisite skills to navigate complex ethical challenges. The findings inform educators, curriculum developers, and policymakers about enhancing ethics education and ensuring the ethical competence of future healthcare practitioners.

## Introduction and background

Ethics education holds profound significance in healthcare, as it equips healthcare professionals and students with the essential competencies to navigate the intricate web of ethical challenges and dilemmas that pervade clinical practice [1]. These ethical predicaments, encompassing conflicts and dilemmas, exert a palpable influence on the daily provision of patient care and treatment. By delving into the dimensions of sensitivity, knowledge, reflection, decision-making, action, and behavior, ethics education cultivates a holistic foundation of ethical competencies that are indispensable for effectively grappling with ethically problematic situations [1,2]. As the torchbearers of the future healthcare workforce, students undergoing training require meticulous instruction to adeptly confront the ethical complexities that await them. Simultaneously, experienced professionals must continually refine their ability to manage and resolve ethical predicaments, ensuring their ongoing growth and development [3]. Ethics education thus assumes a pivotal role in fostering the acquisition and enhancement of ethical competencies among healthcare professionals and trainees, empowering them to address and surmount the multifaceted challenges inherent in their practice.

However, it is crucial to recognize that the study of ethics extends far beyond providing explicit answers or rigid rules of conduct for individual ethical dilemmas. Rather, ethics education offers a framework for cultivating practical wisdom, enabling healthcare professionals to navigate the intricacies of moral quandaries with sagacity and discernment [4]. It serves as a compass, guiding them in defining problems, establishing fundamental principles, and solving ethical conundrums. Moreover, the study of ethics provides a valuable lens through which healthcare professionals can identify appropriate goals and attain clarity of purpose, thereby forging a meaningful trajectory toward their realization. The responsibility of facilitating ethics education falls upon healthcare supervisors, who must provide educational opportunities that nurture professionals capable of taking ethical stands grounded in sound and rational thinking [5]. By fulfilling this duty, individuals within healthcare facilities make invaluable contributions to their profession and society. They cultivate a new generation of healthcare professionals with an unwavering ethical stance fortified by the knowledge, critical thinking skills, and moral integrity nurtured through robust ethics education. In doing so, they forge a path toward a high standard of healthcare practice, one driven by an unwavering commitment to ethical principles and compassionate patient care.

In addition to emphasizing the importance of ethics education in healthcare, it is important to mention that a comprehensive understanding of the field will be furthered by looking at existing research on the topic [6]. Therefore, to shed light on the landscape of ethics education in the research arena, a bibliometric analysis was conducted to explore the scholarly contributions in this domain. Through this analysis, an extensive examination of relevant literature was undertaken, encompassing scholarly articles, research papers, and academic publications. By employing analytical techniques, such as citation analysis, key trends, seminal works, and influential authors in the field of ethics education were identified.

## Review

Methodology

The study examined the 100 most-cited articles related to ethics education, utilizing the SCOPUS engine as the primary data source. The search was performed using predefined search terms, namely, “ethics education,” “medical education,” and “medical ethics education,” to ensure comprehensive coverage of the relevant literature. All types of scientific papers, including original research articles, reviews, and conference papers, were included in the analysis if they pertained to ethics education in healthcare. The articles were systematically assessed, considering various vital parameters for a comprehensive evaluation. These parameters included the journal title, year of publication, total number of citations, citation density, geographic origin, and article type.

The total number of citations received by each article was considered an objective measure of its impact and visibility within the academic community. Additionally, the geographic origin of these papers was examined to ascertain the global distribution of research contributions in ethics education. This analysis provided valuable insights into international collaboration and perspectives in the field. Furthermore, the article type was categorized to identify the diverse scholarly outputs contributing to the ethics education literature. Data extraction and analysis were performed meticulously to ensure accuracy and reliability. Statistical tools were employed to derive meaningful conclusions and identify significant trends within the dataset.

Results

The initial search conducted in the SCOPUS database using the predefined methodology yielded 286 documents. To ensure the inclusion of relevant articles, further refinement was undertaken by combining the research items and narrowing down the search field to focus solely on the “article title.” This step was crucial in aligning the search results with the specific objectives of our study. Considering the diverse linguistic landscape of the retrieved manuscripts, it was observed that translations accompanied all non-English-language articles. We decided not to restrict the search to English-language publications to ensure a comprehensive analysis. This approach allowed us to capture a broader spectrum of literature and gain a more inclusive perspective on ethics education.

Subsequently, a meticulous screening process was conducted to identify the top 100 most-cited articles in accordance with the predetermined criteria. In total, 186 documents were omitted from the analysis, ensuring a focused selection of the most influential and impactful articles in accordance with the top 100 list. The titles of these articles have been presented in Table 1, providing a reference for further examination and analysis.

**Table 1:** The top 100 most-cited articles in ethics education ranked by the number of times cited.

Rank	Title	Authors	Journal	Year	Times cited	Document type
1	The hidden curriculum, ethics teaching, and the structure of medical education	Hafferty et al. [7]	Academic Medicine	1994	1,078	Article
2	Medical ethics education: Where are we? Where should we be going? A review	Eckles et al. [8]	Academic Medicine	2005	217	Review
3	Medical ethics education: Coming of age	Miles et al. [9]	Journal of Medical Education	1989	194	Review
4	Review of ethics curricula in undergraduate medical education	Goldie et al. [10]	Medical Education	2000	141	Review
5	Medical ethics education: Past, present, and future	Fox et al. [11]	Journal of the Association of American Medical Colleges	1995	141	Article
6	Teaching medical ethics and law within medical education: A model for the UK core curriculum. Consensus statement by teachers of medical ethics and law in UK medical schools	Ashcroft et al. [12]	Journal of Medical Ethics	1998	124	Short survey
7	A survey of medical ethics education at U.S. and Canadian medical schools	Lehmann et al. [13]	Academic Medicine	2004	122	Review
8	Ethics education in U.S. medical schools: A study of syllabi	DuBois et al. [14]	Academic Medicine	2002	107	Article
9	The ethics of caring and medical education	Branch WT Jr. [15]	Academic Medicine	2000	104	Article
10	The essential role of medical ethics education in achieving professionalism: The romanell report	Carrese et al. [16]	Academic Medicine	2015	104	Article
11	The effect of team-based learning in medical ethics education	Chung et al. [17]	Medical Teacher	2009	100	Article
12	The positive role of professionalism and ethics training in medical education: A comparison of medical student and resident perspectives	Roberts et al. [18]	Academic Psychiatry	2004	85	Review
13	Perspective: Medical education in medical ethics and humanities as the foundation for developing medical professionalism	Doukas et al. [19]	Academic Medicine	2012	83	Review
14	Context in medical education: The informal ethics curriculum	Hundert et al. [20]	Medical Education	1996	83	Article
15	A randomized trial of ethics education for medical house officers	Sulmasy et al. [21]	Journal of Medical Ethics	1993	71	Article
16	A process evaluation of medical ethics education in the first year of a new medical curriculum	Goldie et al. [22]	Medical Education	2000	53	Article
17	Evaluating ethics competence in medical education	Savulescu et al. [23]	Journal of Medical Ethics	1999	51	Article
18	Medical ethics and law as a core subject in medical education	Doyal et al. [24]	British Medical Journal	1998	50	Editorial
19	Ethics education for medical house officers: Long term improvements in knowledge and confidence	Sulmasy et al. [25]	Journal of Medical Ethics	1997	45	Article
20	Reforming medical education in ethics and humanities by finding common ground with Abraham Flexner	Doukas et al. [26]	Academic Medicine	2010	42	Article
21	The challenge of promoting professionalism through medical ethics and humanities education	Doukas et al. [27]	Academic Medicine	2013	38	Review
22	Internal medicine residents' preferences regarding medical ethics education	Jacobson et al. [28]	Journal of Medical Education	1989	36	Article
23	The ethics of ambiguity: Rethinking the role and importance of uncertainty in medical education and practice	Domen [29]	Academic Pathology	2016	35	Article
24	The ethics of medical education	Jagsi et al. [30]	British Medical Journal	2004	35	Short Survey
25	A data-generated basis for medical ethics education: Categorizing issues experienced by students during clinical training	Bissonette et al. [31]	Academic Medicine	1995	35	Article
26	The role of history and ethics of anatomy in medical education	Hildebrandt [32]	Anatomical Sciences Education	2019	31	Note
27	Transforming educational accountability in medical ethics and humanities education toward professionalism	Doukas et al. [33]	Academic Medicine	2015	29	Review
28	Case-based seminars in medical ethics education: How medical students define and discuss moral problems	Donaldson et al. [34]	Journal of Medical Ethics	2010	29	Article
29	Ethics in medical technology education [Etic în educaţie medicalǎ tehnologicǎ]	Toader [35]	Revista Romana de Bioetica	2010	29	Article
30	Ethics teaching in a medical education environment: preferences for diversity of learning and assessment methods	AlMahmoud et al. [36]	Medical Education Online	2017	28	Article
31	Strengthening the role of ethics in medical education	Singer [37]	Canadian Medical Association Journal	2003	27	Note
32	Why the ethics of medical education research differs from that of medical research	Ten Cate [38]	Medical Education	2009	26	Note
33	Research ethics requirements for medical education	Eva [39]	Medical Education	2009	26	Editorial
34	The program for professional values and ethics in medical education	Lazarus et al. [40]	Teaching and Learning in Medicine	2000	26	Article
35	Compassion as a basis for ethics in medical education	Leget et al. [41]	Journal of Medical Ethics	2007	24	Article
36	Teaching medical ethics: a review of the literature from North American medical schools with emphasis on education	Musick et al. [42]	Medicine, Health Care, and Philosophy	1999	23	Review
37	The adequacy of medical ethics education in a paediatrics training program	Waz et al. [43]	Academic Medicine	1995	23	Article
38	Comparison of lecture and team-based learning in medical ethics education	Ozgonul et al. [44]	Nursing Ethics	2019	22	Article
39	Medical students’ affirmation of ethics education	Lehrmann et al. [45]	Academic Psychiatry	2009	22	Article
40	Understanding, being, and doing: Medical ethics in medical education	Rhodes et al. [46]	Cambridge Quarterly of Healthcare Ethics	2003	21	Review
41	The Duke University program for integrating ethics and human values into medical education	Puckett et al. [47]	Journal of Medical Education	1989	21	Article
42	Addressing medical students’ negative bias toward patients with obesity through ethics education	Geller et al. [48]	AMA Journal of Ethics	2018	20	Article
43	Students’ medical ethics rounds: A combinatorial program for medical ethics education	Beigy et al. [49]	Journal of Medical Ethics and History of Medicine	2016	20	Article
44	The need for medical ethics education in family medicine training	Manson [50]	Family Medicine	2008	20	Review
45	Ethics rounds in a children’s medical center: evaluation of a hospital-based program for continuing education in medical ethics	Levine et al. [51]	Pediatrics	1977	20	Article
46	Teaching medical ethics in graduate and undergraduate medical education: A systematic review of effectiveness	De La Garza et al. [52]	Academic Psychiatry	2017	19	Review
47	Relevance of the rationalist–intuitionist debate for ethics and professionalism in medical education	Leffel et al. [53]	Advances in Health Sciences Education	2014	19	Article
48	The ethics of conducting graduate medical education research on residents	Keune et al. [54]	Academic Medicine	2013	19	Review
49	Two concepts of medical ethics and their implications for medical ethics education	Rhodes [55]	Journal of Medicine and Philosophy	2002	19	Review
50	Ethics approval for research in medical education	Morrison et al. [56]	Medical Education	2001	19	Editorial
51	Bearing response-ability: Theater, ethics and medical education	Rossiter [57]	Journal of Medical Humanities	2012	17	Article
52	Capturing the ethics education value of television medical dramas	White [58]	American Journal of Bioethics	2008	17	Note
53	The globalization of education in medical ethics and humanities: Evolving pedagogy at Weill Cornell Medical College in Qatar	Del Pozo et al. [59]	Academic Medicine	2005	17	Review
54	Ethics of virtual reality in medical education and licensure	Iserson [60]	Cambridge Quarterly of Healthcare Ethics	2018	16	Article
55	Biotechnology and ethics in medical education of the new millennium: Physician roles and responsibilities	Gonnella et al. [61]	Medical Teacher	2001	16	Article
56	Must we remain blind to undergraduate medical ethics education in Africa? A cross-sectional study of Nigerian medical students	Okoye et al. [62]	BMC Medical Ethics	2017	15	Article
57	Using a sledgehammer to crack a nut: Clinical ethics review and medical education research projects	Pugsley et al. [63]	Medical Education	2007	15	Note
58	Ethics in contemporary health care management and medical education	Balak et al. [64]	Journal of Evaluation in Clinical Practice	2020	14	Review
59	Avoiding evasion: Medical ethics education and emotion theory	Leget [65]	Journal of Medical Ethics	2004	14	Article
60	Ethics education for Canadian medical students	Baylis et al. [66]	Academic Medicine	1991	14	Article
61	Publishing ethics in medical education journals	Brice et al. [67]	Academic Medicine	2009	13	Conference Paper
62	Electronic discussion forums in medical ethics education: The impact of didactic guidelines and netiquette	Buelens et al. [68]	Medical Education	2007	13	Article
63	Ethics education at Northwestern University Medical School	Bresnahan et al. [69]	Journal of Medical Education	1989	13	Article
64	Science ethics education: Effects of a short lecture on plagiarism on the knowledge of young medical researchers	Brkic et al. [70]	Journal of B.U.ON.	2012	12	Article
65	Interns’ perceptions on medical ethics education and ethical issues at the Dokuz Eylul University School of Medicine in Turkey	Ozan et al. [71]	Education for Health: Change in Learning and Practice	2010	12	Article
66	Medical ethics education in India	Ravindran [72]	Indian Journal of Medical Ethics	2008	12	Article
67	Medical ethics, education, and the physician’s image	Pellegrino [73]	JAMA: The Journal of the American Medical Association	1976	12	Article
68	The need for health AI ethics in medical school education	Katznelson et al. [74]	Advances in Health Sciences Education	2021	11	Article
69	Ethics curriculum for emergency medicine graduate medical education	Marco et al. [75]	Journal of Emergency Medicine	2011	11	Article
70	Combating junior doctors’ “4 am logic”: A challenge for medical ethics education	McDougall [76]	Journal of Medical Ethics	2009	11	Article
71	Establishment of medical education upon internalization of virtue ethics: Bridging the gap between theory and practice	Madani et al. [77]	Journal of Medical Ethics and History of Medicine	2017	10	Article
72	A theoretical framework for human and veterinary medical ethics education	Magalhães-Sant’Ana [78]	Advances in Health Sciences Education	2016	10	Article
73	Science ethics education part II: Changes in attitude toward scientific fraud among medical researchers after a short course in science ethics	Vuckovic-Dekic et al. [79]	Journal of B.U.ON.	2012	10	Article
74	Medical ethics education: To what ends?	Gross [80]	Journal of Evaluation in Clinical Practice	2001	10	Article
75	The forgotten curriculum: An argument for medical ethics education	Sanders [81]	JAMA	1995	10	Review
76	Medical ethics education	Gillon [82]	Journal of medical ethics	1987	10	Editorial
77	Diversity in approach to teaching and assessing ethics education for medical undergraduates: A scoping review	Souza et al. [83]	Annals of Medicine and Surgery	2020	9	Review
78	Culture and ethics in medical education: The Asian perspective	Shamim et al. [84]	Journal of the Pakistan Medical Association	2018	9	Article
79	Medical ethics education for neurology eesidents: Where do we go from here?	Traner et al. [85]	Seminars in Neurology	2018	9	Article
80	Using Kantian ethics in medical ethics education	Donaldson et al. [86]	Medical Science Educator	2017	9	Note
81	Teaching corner: An undergraduate medical education program comprehensively integrating global health and global health ethics as core curricula: Student experiences of the Medical School for International Health in Israel	Teichholtz et al. [87]	Journal of Bioethical Inquiry	2015	9	Article
82	Deliberation at the hub of medical education: Beyond virtue ethics and codes of practice	Barilan et al. [88]	Medicine, Health Care and Philosophy	2013	9	Article
83	Science ethics education. Part I. Perception and attitude toward scientific fraud among medical researchers	Vuckovic-Dekic et al. [89]	Journal of B.U.ON.	2011	9	Article
84	Strategies for incorporating professional ethics education in graduate medical programs	Bolin [90]	American Journal of Bioethics	2006	9	Note
85	The importance of including bio-medical ethics in the curriculum of health education institutes	Selvakumar et al. [91]	Education for Health	2004	9	Article
86	Postgraduate education in medical ethics in Japan	Asai et al. [92]	Medical Education	1998	9	Article
87	A systematic scoping review of undergraduate medical ethics education programs from 1990 to 2020	Wong et al. [93]	Medical Teacher	2022	8	Article
88	The state of ethics education at medical schools in Turkey: Taking stock and looking forward	Kavas et al. [94]	BMC Medical Education	2020	8	Article
89	Ethics competences in the undergraduate medical education curriculum: The Spanish experience	Ferreira-Padilla et al. [95]	Croatian Medical Journal	2016	8	Article
90	How not to think: Medical ethics as negative education	Cigman [96]	Medicine, Health Care and Philosophy	2013	8	Article
91	Systematic review of ethics consultation: A route to curriculum development in post-graduate medical education	Mueller et al. [97]	American Journal of Bioethics	2006	8	Note
92	Psychiatry’s contribution to medical ethics education	Sider et al. [98]	American Journal of Psychiatry	1982	8	Article
93	Preclinical students’ views on medical ethics education: A focus group study in Turkey	Bilgin et al. [99]	Acta Bioethica	2018	7	Article
94	Medical ethics education in china: Lessons from three schools	Sherer et al. [100]	Education for Health: Change in Learning and Practice	2017	7	Article
95	Research ethics and medical education	Hally et al. [101]	Medical Teacher	2016	7	Letter
96	Medical ethics, bioethics and research ethics education perspectives in South East Europe in graduate medical education	Mijaljica [102]	Science and Engineering Ethics	2014	7	Article
97	Medical ethics education: A survey of opinion of medical students in a Nigerian University	Ogundiran et al. [103]	Journal of Academic Ethics	2010	7	Article
98	Ethics in medical education [Ethik in der Medizinischen Aus- und Weiterbildung]	Neitzke et al. [104]	Bundesgesundheitsblatt - Gesundheitsforschung - Gesundheitsschutz	2008	7	Article
99	Requirements for ethics, socioeconomic, and legal education in postgraduate medical programs.	Iserson et al. [105]	The Journal of Clinical Ethics	1993	7	Article
100	Review of ethics teaching in undergraduate medical education	Shamim et al. [106]	Journal of the Pakistan Medical Association	2020	6	Review

Citation count, document types, and geographical origin

**Figure 1:**
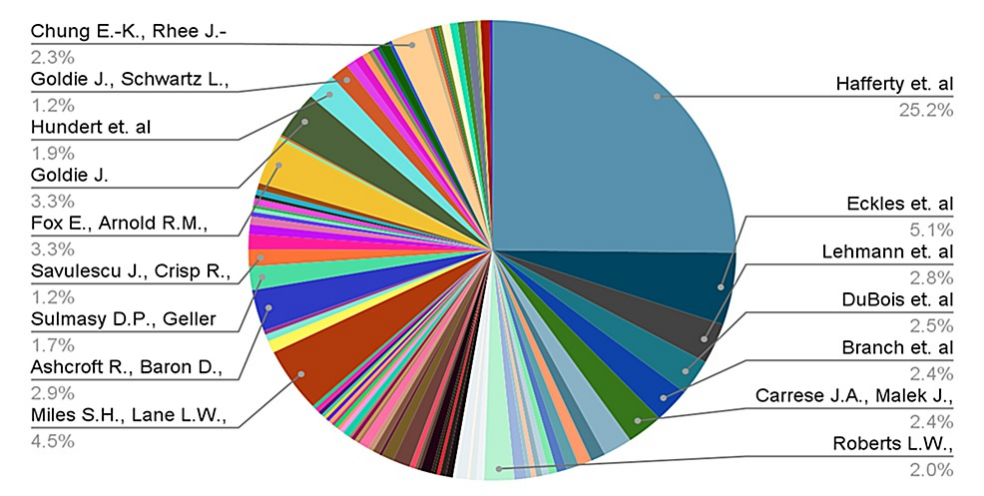
Pie chart representing the number of citations associated with author names. [7-18,20,22,23].

Collectively, these aforementioned articles amassed a total of 4,284 citations, demonstrating the widespread recognition and enduring influence of the selected publications. On average, each article received 42 citations (mean ± SD = 42.84 ± 11.18). Figure 1 provides a visual representation of the citation counts for the top 100 articles in the ethics education field. Notably, the publication by Hafferty and Frank, titled “The hidden curriculum, ethics teaching, and the structure of medical education,” published in Academic Medicine (1994), emerges as a standout with the highest number of citations [7].

**Figure 2:**
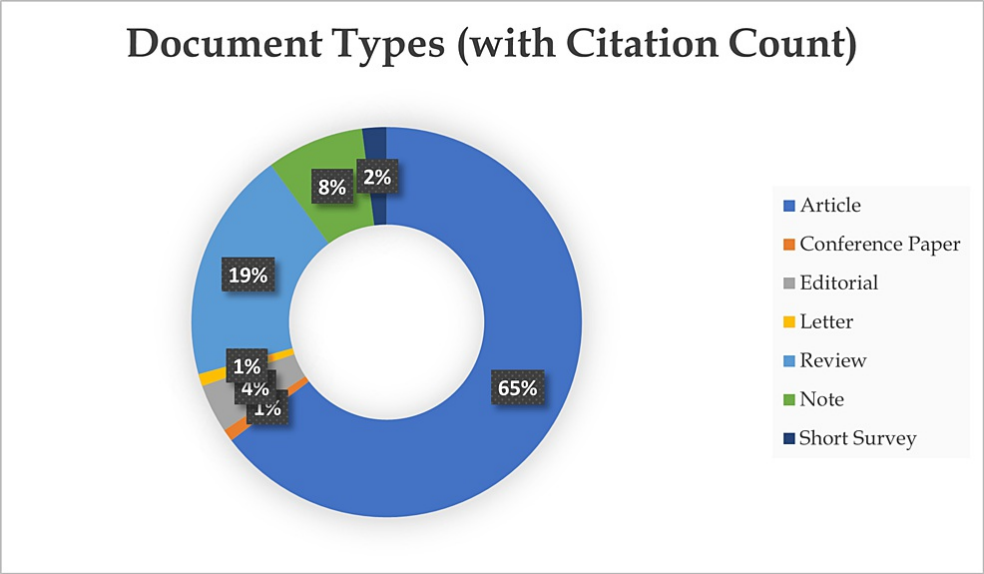
Donut chart representing the various types of publications along with their respective quantities.

The analysis of document types within these top 100 articles revealed distinct patterns in scholarly contributions (Figure 2). The majority of articles, comprising 65% of the dataset, were classified as original articles. These original research articles signified primary investigative endeavors and empirical studies conducted in the field of ethics education, highlighting the commitment to advancing knowledge and understanding. Additionally, 19% of the articles were identified as review articles. These comprehensive literature reviews synthesized existing research, critically analyzing and consolidating knowledge on specific aspects of ethics education. Within the remaining document types, notes and letters were identified as separate categories in the SCOPUS database. However, it is important to note that most journals typically classify them as either “letters” or “comments.” Together, these shorter forms of communication constituted 9% of the overall dataset.

Regarding geographical origin, the majority of the selection of the top 100 cited papers originated from the United States, thus accentuating the substantial presence and impact of American scholars within the realm of ethics education. The United Kingdom emerged as the second most prominent contributor to the body of literature on ethics education, and a perusal of relevant titles demonstrates how British researchers’ focus on ethics education resonates with the emphasis placed on professionalism and the ethical facets of healthcare practice.

**Figure 3:**
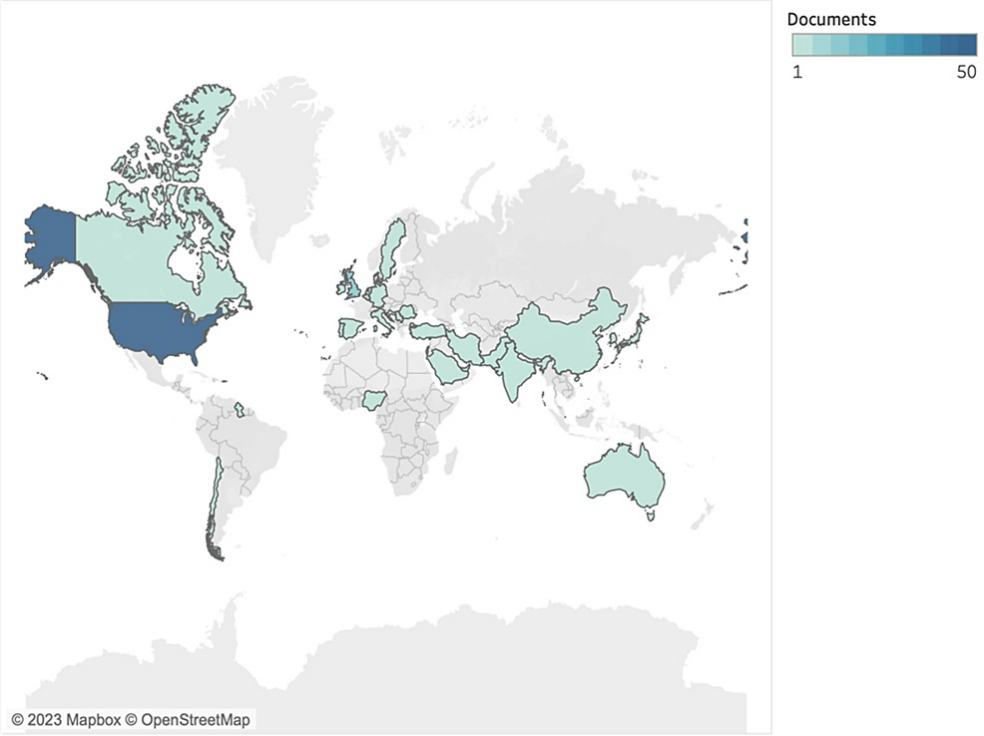
Document density for the top 100 articles based on countries.

In recent years, there has also been a noticeable rise in interest in and engagement with ethics education in countries such as Turkey, India, and Pakistan. These regions have grown committed to incorporating ethics education into their medical and healthcare curricula, illustrating the global relevance and impact of ethics education in diverse cultural contexts (Figure 3).

Journal analysis and publication year

The analysis of the top 100 articles in ethics education revealed a notable concentration of publications in a select number of journals (Table 2). Among the identified journals, Academic Medicine emerged as the leading publication, with 16 papers included in the top 100. It is noteworthy to mention that the Journal of Medical Education, although now known as Academic Medicine, was considered separately in this analysis due to the fact that the original articles were published under that name. This distinction is maintained to acknowledge the historical context and ensure accuracy in referencing the original articles. Additionally, the Journal of Medical Ethics and Medical Education both had nine papers, each within the top 100 articles, reflecting their substantial contributions to the ethical dimensions of healthcare education. Medical Teacher also featured prominently, with four papers included in the top 100. This journal specializes in medical education, offering a platform for sharing innovative teaching methods and pedagogical approaches.

**Table 2:** Journals appearing in the top 100 list.

Rank	Journal	Number of articles
1	Academic Medicine	16
2	Journal of Medical Ethics	9
3	Medical Education	9
4	Journal of Medical Education	4
5	Medical Teacher	4
6	Medicine, Health Care & Philosophy	3
7	Academic Psychiatry	3
8	British Medical Journal (BMJ)	2
9	Education for Health: Change in Learning and Practice	3
10	Advances in Health Sciences Education	3
11	Journal of B.U.ON.	3
12	American Journal of Bioethics	3
13	Cambridge Quarterly of Healthcare Ethics	2
14	Journal of Medical Ethics and History of Medicine	2
15	Journal of Evaluation in Clinical Practice	2
16	JAMA	2
17	Journal of the Pakistan Medical Association	2
18	AMA Journal of Ethics	1
19	Teaching and Learning in Medicine	1
20	Family Medicine	1
21	Paediatrics	1
22	Anatomical Sciences Education	1
23	Journal of Medicine & Philosophy	1
24	Journal of Medical Humanities	1
25	Medical Education Online	1
26	BMC Medical Ethics	1
27	Nursing Ethics	1
28	Revista Romana de Bioetica	1
30	Indian Journal of Medical Ethics	1
31	Journal of the Association of American Medical Colleges	1
32	Journal of Emergency Medicine	1
33	Annals of Medicine & Surgery	1
34	Canadian Medical Association Journal	1
35	Seminars in Neurology	1
36	Medical Science Educator	1
37	Journal of Bioethical Inquiry	1
38	Academic Pathology	1
39	BMC Medical Education	1
40	Croatian Medical Journal	1
41	American Journal of Psychiatry	1
42	Acta Bioethica	1
43	Science and Engineering Ethics	1
44	Journal of Academic Ethics	1
45	Bundesgesundheitsblatt - Gesundheitsforschung - Gesundheitsschutz	1
46	The Journal of Clinical Ethics	1

Notably, four out of the five analyzed journals, namely, Academic Medicine, Medical Education, Journal of Medical Education (now Academic Medicine), and Medical Teacher, are specifically focused on medical education. This highlights the central role of educational strategies, curriculum development, and pedagogical considerations in the ethical development of healthcare professionals. The Journal of Medical Ethics, although not primarily focused on medical education, emerged as a significant contributor emphasizing ethical issues and healthcare debates. The inclusion of this journal reflects the broader perspective it provides in the ethical discourse, enriching the understanding of ethical considerations beyond educational aspects.

The analysis of the publication years of the top 100 articles in ethics education reveals an uptrend in research output from the year 2000 onward (Table 3). During this period, 33 articles were published, indicating a surge in scholarly activity and a growing emphasis on ethics education in healthcare. This increase in publications reflects a heightened recognition of the importance of ethical considerations in healthcare practice and the need to equip healthcare professionals with the necessary ethical competencies. Therefore, it signifies a collective effort to address the ethical challenges and dilemmas that arise in providing healthcare services.

**Table 3:** Decade of publication for articles in the Top 100 list

Decade of publication	Number of articles
1970–1979	2
1980–1989	6
1990–1999	15
2000–2009	33
2010–2019	38
2020–Present	6

Interestingly, while the uptrend in research output is observed from 2000 onward, the most-cited paper within the top 100 articles dates back to 1994. This indicates that despite the subsequent increase in publications, the influence and impact of this seminal work by Hafferty and Frank have endured over time. It remains a cornerstone publication in the field, guiding and shaping the discourse on ethics education in healthcare.

Author analysis and keyword usage

The analysis of authors within the top 100 articles in ethics education provides insights into the individuals who have made significant contributions to the field (Table 4). David J. Doukas, a bioethicist based at Tulane University [107], is recognized as one of the most influential authors in the field of ethics education, with five articles in the top 10 list attributed to his name that have amassed a total of 296 citations. Similarly, Laurence B. McCullough, another prominent figure in the field, is also credited with five articles within the top 100 and a total citation count of 286. Interestingly, McCullough shares four of his top 10 cited papers with Doukas, indicating mutual exploration of similar research themes [16,19,26,27].

**Table 4:** Top 11 authors appearing in the top 100 list.

Author name	Number of articles (n)
David J. Doukas	5
Laurence B. McCullough	5
Lisa Soleymani Lehmann	4
Stephen Wear	4
Silvija Brkic	3
Gordana M Bogdanovic	3
Ljiljana Vučković-Dekić	3
Iva Kezic	3
Dušica Gavrilović	3
Gail Geller	3
Raanan Gillon	3

**Figure 4:**
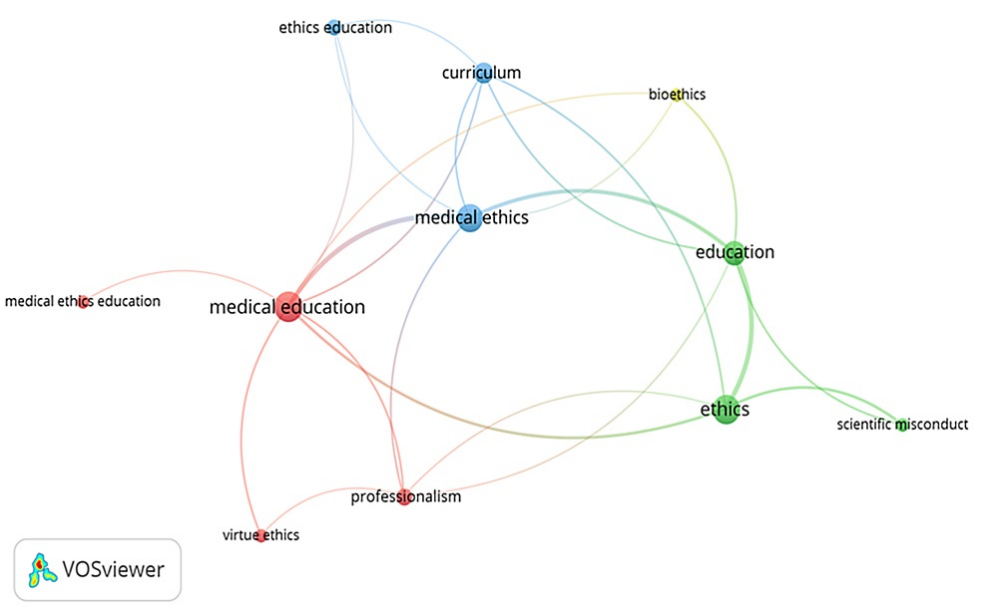
Network analysis of author keywords from the top 100 list.

The author keywords of all 100 articles were subjected to a comprehensive analysis using VOSviewer network analysis, as illustrated in Figure 4. This analysis aimed to identify the main keywords used by the authors and gain insights into the prevailing themes in ethics education. Notably, keywords such as “medical education,” “medical ethics,” “education,” “curriculum,” “ethics,” and “professionalism” were identified as central themes within the field of ethics education.

Discussion

Ethics education in healthcare is a critical component of medical training, aiming to equip healthcare professionals with the necessary ethical competencies to navigate complex ethical dilemmas. In this study, we examined the 100 most-cited articles related to ethics education in healthcare, analyzing various parameters such as citation count, document types, geographical origin, journal analysis, publication year, author analysis, and keyword usage.

The analysis of the citation count revealed that the selected articles had a significant impact on the academic community, with a total of 4,284 citations. The most cited article, “The hidden curriculum, ethics teaching, and the structure of medical education” by Hafferty and Frank, published in Academic Medicine in 1994, stands out as the most influential publication in the dataset [7]. This seminal work continues to shape the discourse on ethics education, highlighting the importance of addressing the hidden curriculum and the structure of medical education in the ethical development of healthcare professionals. Similarly, analysis of the publication years revealed an increasing research output in ethics education from the year 2000 onward. This surge in scholarly activity indicates a growing recognition of the importance of ethics education in healthcare and a concerted effort to address the ethical challenges healthcare professionals face. It also reflects the evolving nature of healthcare practice, with ethical considerations becoming more prominent in the delivery of healthcare services.

At the same time, a closer look at journal publications demonstrates a concentration of articles in a select number of journals, primarily focusing on medical education and ethics. This concentration highlights the central role of educational strategies, curriculum development, and pedagogical considerations in ethics education. It also emphasizes the need for collaboration between the fields of medical education and ethics to ensure the integration of ethical principles and practices into healthcare professional training. Furthermore, our study also examined prominent contributors in the field of ethics education, with David J. Doukas and Laurence B. McCullough emerging as influential figures. Their contributions signify their expertise and dedication to advancing knowledge in ethics education. The presence of common themes explored by these authors suggests a shared interest in and collaborative exploration of specific research areas, further enriching the discourse on ethics education.

In addition to the parameters analyzed in the previous discussion, it is important to note that a significant proportion of the top 100 articles in ethics education focused on undergraduate medical students. These articles recognized the importance of starting ethics education early in medical training to shape the ethical development of young minds. Ethics education for undergraduate medical students is crucial as it lays the foundation for the ethical principles and values that guide their professional practice throughout their careers. By introducing ethics education early, students can develop a strong ethical framework, enhance their moral reasoning skills, and cultivate a sense of professionalism.

Interestingly, analysis of the newer articles (2015 onward) reveals a shift in focus toward themes such as virtual reality and artificial intelligence [60,74]. These emerging topics highlight the evolving landscape of healthcare education and the ethical considerations that arise with integrating new technologies.

Regrettably, simulation-based education, despite its increasing popularity and relevance in healthcare education, seems to be comparatively underrepresented within the dataset. Nonetheless, it offers an exceptional platform for ethics education as it creates a safe and controlled environment for students to delve into and navigate intricate ethical dilemmas. Learners can significantly enhance their ethical competence by actively engaging in decision-making, ethical reasoning, and communication skills. Simulated scenarios also provide a unique opportunity for students to develop and refine their ethical frameworks, clinical judgment, and interprofessional collaboration, ultimately equipping them to tackle the ethical challenges they will inevitably face in their future clinical practice [108].

## Conclusions

Overall, this study sheds light on the current landscape of ethics education by analyzing the most-cited articles in the field. It identifies emerging themes, highlights the importance of simulation-based education, and emphasizes the need for interdisciplinary collaboration in ethics education. The findings of this study can inform educators, curriculum developers, and policymakers in designing and implementing effective ethics education programs that prepare future healthcare professionals to navigate complex ethical challenges. Further research and exploration in these areas are warranted to continue advancing ethics education and ensuring the ethical competence of healthcare professionals.
